# Bell's palsy during rechallenge of immune checkpoint inhibitor

**DOI:** 10.1002/iju5.12572

**Published:** 2023-01-16

**Authors:** Kohji Takemura, Taro Yamanaka, Michikata Hayashida, Rika Kizawa, Takeshi Yamaguchi, Yuko Tanabe, Kazushige Sakaguchi, Koichi Suyama, Shinji Urakami, Yuji Miura

**Affiliations:** ^1^ Department of Medical Oncology Toranomon Hospital Tokyo Japan; ^2^ Department of Urology Toranomon Hospital Tokyo Japan

**Keywords:** Bell's palsy, immune‐related adverse event, nivolumab, peripheral facial nerve palsy, rechallenge of immune checkpoint inhibitor

## Abstract

**Introduction:**

The peripheral nervous system is one of the target organs of immune‐related adverse events. Peripheral facial nerve palsy, also called Bell's palsy, which is induced by immune checkpoint inhibitors, is quite rare, and its clinical features are not well known.

**Case presentation:**

A man with renal cell carcinoma who received rechallenging immune checkpoint inhibitor therapy developed unilateral facial palsy and was diagnosed with Bell's palsy. He did not have any severe immune‐related adverse events during his previous immune checkpoint inhibitor treatment. Corticosteroid therapy was immediately initiated, and his facial palsy symptoms promptly improved.

**Conclusion:**

Physicians should be aware that Bell's palsy can occur as an immune‐related adverse event. Additionally, careful observation is necessary during rechallenge with immune checkpoint inhibitors, even in patients who did not have previous immune‐related adverse events.

**Keynote message**

Careful attention should be paid to Bell's palsy during treatment with immune checkpoint inhibitors, even if the previous treatment was safe. When suspected, it is essential to promptly perform a neurological evaluation and initiate appropriate treatment.

**Abbreviations & Acronyms**
ccRCC: clear cell renal cell carcinoma
ICI: immune checkpoint inhibitor
IMDC: International Metastatic Renal Cell Carcinoma Database Consortium
irAE: immune‐related adverse event
MRI: magnetic resonance imaging
PD‐1: programmed cell death 1

## Introduction

ICI has significantly improved the prognosis of various cancer types. ICI can cause various types of irAEs. Although neurologic irAEs occur infrequently, it is important to be aware of them because of their potential risk of severity. In a literature review, the overall incidence of neurologic irAEs with anti‐PD‐1 antibodies is 6.1%, and the incidence of severe neurologic irAEs is <1%.^1^ Its spectrum is broad and includes encephalopathy, Guillain‐Barre‐like syndrome, peripheral neuropathy, and myasthenic syndrome. Previous case reports have shown that Bell's palsy is a spectrum of neurological irAEs. However, its frequency is extremely low, and its clinical features are not well known.^2^

## Case presentation

**Fig. 1:**
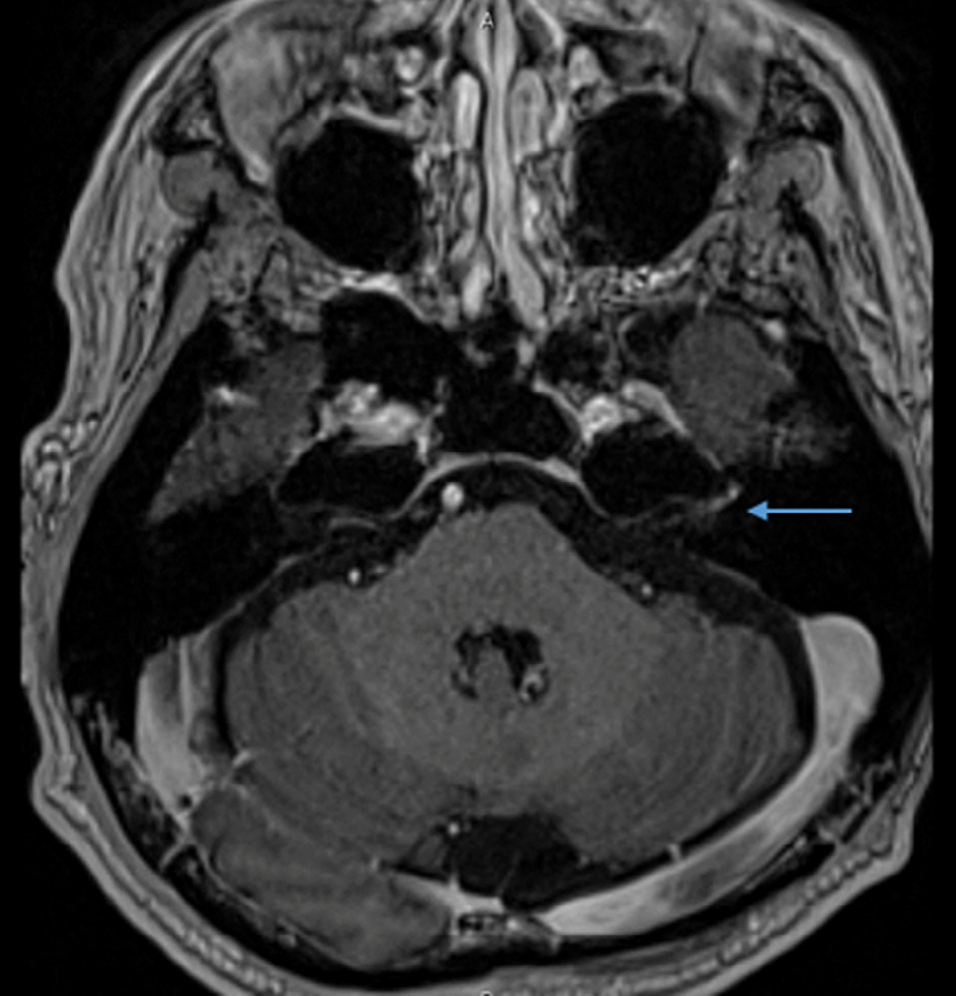
T1 weighted MRI image with contrast medium. The blue arrow shows the enhancement of the left facial nerve.

A 55‐year‐old man was diagnosed with ccRCC with metastasis to the lungs, liver, bone, and multiple lymph nodes. The IMDC risk category was favorable. The patient received a combination of axitinib and pembrolizumab as a first‐line therapy, with the greatest effect of partial response and without severe irAEs. The treatment was discontinued after 34 cycles, owing to disease progression. Subsequently, everolimus and then cabozantinib were administered to the patient. Subsequently, nivolumab was started as a rechallenge for ICI therapy. On the eleventh day after the first administration, the patient had difficulty closing the left eyelid and mouth fully. He visited our hospital 4 days after onset, and a physical examination revealed paralysis of the upper and lower left facial muscles, suggesting peripheral facial nerve paralysis. Facial paresthesia, hearing loss, dizziness, tinnitus, painful rash on the auricle, dysphagia, and dysarthria were not observed. There were no abnormalities in other cranial nervous systems. Contrast‐enhanced cranial MRI detected abnormal enhancement in the left facial nerve (Fig. 1), but no brain metastasis or cerebral infarction was noted. The serological antigen test was negative for herpes simplex virus and varicella zoster virus. The patient had no history of diabetes mellitus or neurological disorders. Since other causes could be excluded based on the clinical course, physical examination, blood tests, and imaging tests, the patient was diagnosed with Bell's palsy, most suspiciously induced by nivolumab. Treatment with oral prednisone equivalent to 0.5 mg/kg/day was started. Facial weakness improved promptly in 4 days, and prednisone was completed in 14 days. After 2 months, re‐examination with cranial MRI revealed the residual contrast enhancement in the left facial nerve despite complete resolution of paralytic symptoms. We permanently discontinued nivolumab and started subsequent anticancer therapy because rapid disease progression of hilar lymph node metastasis was confirmed.

## Discussion

We encountered a case of peripheral facial nerve palsy with rapid onset after the initiation of nivolumab therapy, which was finally diagnosed as Bell's palsy most likely induced by nivolumab. However, it is necessary to properly exclude the differential diagnosis, and a few important points should be considered. First, many possible pathologies can cause facial nerve palsy, such as viral infection due to herpes simplex virus and varicella zoster virus; brain tumors, including metastasis; autoimmune diseases, such as Guillain‐Barre syndrome; cerebrovascular diseases, and diabetes mellitus.^3^ The current case showed weakness in both the upper and lower facial muscles, consistent with peripheral facial nerve palsy.^4^ Further investigations by neurological examination, blood test, and MRI revealed no abnormalities in other nervous systems and excluded other differential diagnoses of facial nerve palsy. Therefore, a diagnosis of peripheral facial nerve palsy, so‐called Bell's palsy, was made. Second, the association between the symptoms and nivolumab therapy should be considered. We thought it possible to diagnose this case as immune‐related Bell's palsy since this case did not contradict the clinical course of previously reported cases, including pembrolizumab,^5^ atezolizumab,^6^ and a combination of nivolumab and ipilimumab^7^, ^8^ although the possibility of idiopathic Bell's palsy that occurred incidentally could not be completely ruled out. According to these reports, the initial symptoms are unilateral facial paralysis and numbness. The typical onset time is several days or weeks after the initiation of ICI therapy. Abnormal enhancement of the unilateral facial nerve can be detected on contrast‐enhanced MRI; however, plain MRI is not useful. Compared to these previous reports, there was a lack of numbness in our case. The positive enhancement on MRI disappeared after symptom improvement in one report,^5^ but not in our case. The condition responded to corticosteroid treatment, and the symptoms improved in all cases. The initiation of corticosteroids within 72 h of symptom onset may be essential for patients with irAE Bell's palsy according to the recommendation of the treatment for idiopathic Bell's palsy.^3^, ^9^

Notably, the current patient developed irAE‐suspected Bell's palsy during the rechallenge of ICI treatment. He had developed no severe irAEs during the previous treatment with pembrolizumab for approximately 2 years, but nivolumab was administered only once, despite an anti‐PD‐1 antibody with a similar mechanism of action as that of pembrolizumab, which induced immune‐related neurotoxicity. The efficacy and safety of ICI rechallenge are still unclear. In a retrospective study,^10^ the response rate of the second ICI for renal cell carcinoma was 23%, although 72% of the patients discontinued the first ICI due to disease progression. Therefore, ICI rechallenge can be a treatment option for selected patients in clinical practice. Conversely, physicians should be careful about irAEs during the rechallenge of ICI, even in patients without irAEs in the first ICI.^10^ In terms of neurological irAEs, according to an observational cohort study that evaluated the rechallenge of ICI after the development of irAEs, the rate of neurological irAE recurrence after ICI rechallenge (6%) was relatively lower than that of other types of irAEs.^11^

In conclusion, physicians should consider Bell's palsy as an irAE. When readministering ICI, it is necessary to pay attention not only to the relapse of irAEs but also to the onset of another irAE. The biological mechanism and pathogenesis of immune‐related Bell's palsy remain to be fully understood, and further studies are warranted to reveal the details of ICI‐related neurotoxicity.

## Author contributions

**Kohji Takemura:** Data curation; investigation; visualization; writing – original draft. **Taro Yamanaka:** Investigation; writing – review and editing. **Michikata Hayashida:** Writing – review and editing. **Rika Kizawa:** Writing – review and editing. **Takeshi Yamaguchi:** Writing – review and editing. **Yuko Tanabe:** Writing – review and editing. **Kazushige Sakaguchi:** Writing – review and editing. **Koichi Suyama:** Writing – review and editing. **Shinji Urakami:** Writing – review and editing. **Yuji Miura:** Conceptualization; writing – review and editing.

## Conflict of interest

The authors declare no conflict of interest. Except for Yuji Miura: personal fees from Ono Pharmaceutical, Bristol Myers Squibb, MSD, and Takeda; Advisory Board personal member of Chugai Pharmaceutical Co. and Takeda; Local PI and Institutional Financial interest from MSD and Ono Pharmaceutical. All of them are outside the submitted work.

## Approval of the research protocol by an Institutional Reviewer Board

Not applicable.

## Informed consent

Written informed consent was obtained from the patient for publication of this case report.

## Registry and the Registration No. of the study/trial

Not applicable.
